# The Influence of Authentic Leadership Perception on Clinical Nurses' Voice Behaviour and the Mediating Effect of Conscientiousness

**DOI:** 10.1155/2024/9946881

**Published:** 2024-02-17

**Authors:** YuJun Fan, Xiaoyuan Qu, Wuxing Zhang, Zhimin Tao, Chaoran Chen

**Affiliations:** ^1^School of Nursing and Health, Henan University, Kaifeng, China; ^2^Institute of Nursing and Health, School of Nursing and Health, Henan University, Kaifeng, China

## Abstract

**Background:**

Clinical nurses, being integral members of the medical system and actively engaged with patients and their families, possess significant influence in addressing various work-related issues and contribute significantly to the advancement of clinical services and the overall stability of hospitals within the nursing team. Consequently, it is imperative to prioritize the consideration of nurses' recommendations in order to identify the factors that can effectively enhance their enthusiasm in vocalizing their concerns.

**Design:**

Data in this cross-sectional descriptive study were collected from March 2021 to August 2021 by the online survey method.

**Methods:**

A total of 679 Chinese nurses were surveyed with a Chinese Big Five Personality Inventory Brief Scale, an Authentic Leadership Scale, and a Voice Behaviour Scale. Because the data were normally distributed in our study, Pearson's correlation coefficient (*r*) was used to conduct the correlation analysis of the study variables. The structural equation model was used to examine the mediating role of conscientiousness.

**Results:**

The results showed that the influencing factors of nurses' voice behavior were education background, employment mode, nursing seniority, and monthly income (*p* < 0.05). In addition, authentic leadership, conscientiousness, and voice behaviour were significantly positively correlated (the correlation coefficients are 0.632 and 0.630, respectively, *p* < 0.05). Conscientiousness plays a partial mediating role in authentic leadership.

**Conclusion:**

Authentic leadership was the key to improving the voice behaviour of nurses; as a mediating mechanism, conscientiousness further explained how authentic leadership promoted the voice behaviour of nurses. The effects of authentic leadership, conscientiousness, and voice behaviour could be used to guide the management of clinical nurses. In particular, the authentic leadership style perceived by nurses and the conscientiousness of nurses would contribute to the generation of voice behaviour.

## 1. Introduction

In today's complex and rapidly developing medical environment, nurses tend to be the main providers of primary health care services, and the central role of nurses in health care, including their proximity to patients, clinical decision-making roles, health care education, and preventive services [1–3]. Nurses not only have a significant voice in work-related issues but also play a crucial role in advancing clinical services and maintaining hospital stability [4]. In order to promote the development of the organization, the team hopes that members can actively provide opinions and suggestions, rather than just completing their own work, which needs to mobilize the enthusiasm of subordinates to participate in the day-to-day management of the organization [5–7]. Voice behaviour can be used as one of the ways for members to participate in organizational management, which can not only enhance the protagonist awareness of subordinates but also give full play to their subjective initiative [8].

Recent studies have demonstrated that managers greatly influence employee voice behaviour, as the primary recipients [9]. Authentic leadership is the concept that managers shape their subordinates' knowledge, attitudes, and behaviours through their genuine qualities and actions in the management process [10]. And the authentic leader focuses on the positive role modelling of honesty, authenticity, and high ethical standards in the development of leader-follower relationships [11]. This type of leadership is characterized by self-awareness, internalized moral perspective, balanced processing of information, and relational transparency, which can affect employees, stimulate employees' authenticity, enhance their trust in their superiors, and thus promote the occurrence of voice behaviour [10].

Therefore, this study proposed the following hypothesis:

  Hypotheses 1. Authentic leadership of nurse managers has a direct positive effect on nurses' voice behaviour.

In addition to the influence of some external environment, employees' personal factors, such as personality characteristics, are also a factor affecting their voice behaviour. An academic study by Wechsler has shown that individual personality, as a kind of tendency, can dominate people's behaviour and perception [12]. For example, the introverted personality tends to look for reasons within itself, has a strong introspection, likes to be alone, and avoids more social contact [13]. The personality of the external control type usually believes that the occurrence of things is affected by external factors, and people with this personality prefer to communicate with others [14]. In other words, personality traits, as the expression of the individual's internal character, will have a more important impact on the individual's psychological state and behavioural performance through different attribution methods. This finding is similar to the attribution theory, which holds that individuals have a process of interpretation and judgment of how events or behaviours occur, and these processes will further affect people's subsequent attitudes and behaviours [15–17].

The big five personality model is currently the most widely used personality trait model to make up the personality structure, including neuroticism, conscientiousness, agreeableness, openness, and extraversion [12]. It has been shown to effectively predict individual work behaviour [18]. Relevant literature has shown that different dimensions of big five personality have different predictive effects of employees' voice behaviour. Lepine et al. [6] revealed that the four dimensions of big five personality have a significant relationship with voice behaviour, among which conscientiousness and openness have a positive impact on employees' voice behaviour. In other words, employees with high conscientiousness have stable emotional attachments with the organization, and they tend to show more positive voice behaviours for the organizational interests. Based on the above statements, the following hypothesis is proposed:

  Hypotheses 2. Conscientiousness has a direct positive effect on nurses' voice behaviour.

After systematically organizing the research on personality traits and employees' voice behaviour, Yanzhe et al. [19] found that among the big five personality traits, conscientiousness and extraversion can positively predict employees' voice behaviour. At the same time, some studies also found that the influence of personality traits on voice behaviour mainly acts through motivation, interpersonal relationships, and emotional mechanisms, and also through the personal characteristics and leadership behaviour of leaders [20–22]. Therefore, when nurses are faced with authentic leadership, whether the influence of this type of leadership on their voice behaviour can be changed to a certain extent through the interpretation and influence of different personality traits remains to be explored. Thus, the following hypotheses were developed:

  Hypotheses 3. Conscientiousness plays a mediating role between authentic leadership and voice behaviour.

Understanding the ways in which the authenticity of nurse leaders could promote nurses' voice behaviour is important. In this essence, in this study, we developed a conceptual model relating authentic leadership of nurse managers to nurses' voice behaviour. In this model, it is anticipated that conscientiousness would mediate the link between the authentic leadership of nurse managers and nurses' voice behaviour.

To sum up, this study was designed to (1) investigate the relationship between the authentic leadership of nurse managers and nurses' voice behaviours, (2) examine the relationship between conscientiousness of nurse and nurses' voice behaviour, and (3) explore the mediating role of nurses' conscientiousness in the relationship between nurse managers' authentic leadership and nurses' voice behaviour.

## 2. Methods

### 2.1. Study Design and Sample

A cross-sectional study was adopted, and the method of convenience sampling was used in this study. Participants in this study were nurses from five general hospitals in north China. Data collection was conducted from March 2021 to August 2021.

A standardized questionnaire will be made and distributed to our subjects (clinical nurses). Before the investigation, researchers were trained in a unified manner. And all of the investigators used a unified guidance language to avoid leading statements. Before the survey, participants were informed of the purpose and significance of the survey and the precautions during the survey. All participants were anonymous and voluntary. The effectiveness of the questionnaire was ensured through an on-site audit and the audit on the same day. Researchers conducted a one-to-one survey of nurses. During the survey, participants were asked to think of a specific leader while filling out questionnaires.

Because this study is descriptive and the primary outcome is a continuous variable, the sample size was estimated using the following formula [23]:

(1) $$\begin{array}{c} n={\left(\frac{U\alpha /2\sigma }{\delta }\right)}^{2}={\left(\frac{1.96\times 1.80}{2}\right)}^{2}=553.19\approx 554\text{ nurses}, \end{array}$$

where *n* indicates the required sample size, *U*_*α*/2_ is the standardized normal deviation corresponding to *α* = 0.05 and 95% confidence level (*U*_*α*/2_ = 1.96, for two-tailed), *σ* indicates the expected value of standard deviation in the population (*σ* = 1.80 from the pilot study), and *δ* indicates the acceptable margin of error for the mean (*δ* = 0.2, based on the pilot study). To consider the nonresponse rate and uncompleted questionnaires, 700 nurses were invited to take part in the study.

The inclusion criteria were as follows: (a) licensed staff nurses; (b) nurses on duty during the study period; and (c) nurses had no less than 1 years of tenure in their current hospital. And the exclusion criteria were the rotation of nurses, or those who were unable to participate in the study due to vacation, leave, and illness. Of the 700 questionnaires returned, 21 were invalid and excluded from the analysis. Thus, the final sample size for this study was 679 with an effective rate of 97%.

### 2.2. Instruments

#### 2.2.1. Sociodemographic Data Questionnaire for Clinical Nurses

After reviewing the literature, we designed the sociodemographic data questionnaire for clinical nurses based on the needs of this study, including their gender, age, marital status, education, hiring method, nursing tenure, and monthly income.

And this study also used three standardized scales to collect the data. And the scales used in this study were professionally translated into Chinese. All items were measured on a 5-point Likert scale.

#### 2.2.2. Authentic Leadership

This study adopts the authentic leadership questionnaire developed by Walumbwa et al. [24], which tests the sample data of Chinese enterprises in the development process of the questionnaire. And this is the most widely used survey scale on authentic leadership at present. The scale includes 4 dimensions, including 4 items of self-awareness, 5 items of relational transparency, 4 items of internalized moral, and 3 items of balanced processing, for a total of 16 items. The Cronbach *α* value of the scale was 0.95.

#### 2.2.3. Conscientiousness

This study adopts the Chinese Big Five Personality Inventory brief version (CBF-PI-B) compiled by Mengcheng Wang and Xintong et al. on the basis of the China Big Five Personality Questionnaire [25], including five dimensions: neurotic, conscientiousness, agreeableness, openness, and extroversion. There are 8 questions for each personality trait. The Cronbach *α* value of the scale was 0.93. And this study had selected questions about conscientiousness.

#### 2.2.4. Voice Behaviour

The 10-item Chinese version of the voice behaviour scale was developed by Liang et al. [26]. The tool consisted of two dimensions: promotive voice and prohibitive voice. The Cronbach *α* value of the scale was 0.93.

### 2.3. Pilot Study

To ensure the questionnaire was understandable and clear, we did a pilot study. And in the pilot study, we also estimated a preliminary mean of the outcome variables to estimate the sample size needed. We recruited 30 nurses from the participating hospitals, in the pilot study, who were then excluded from the study sample. The questionnaire took approximately 5–10 min to complete, and piloted nurses assured that the items were understandable and clear.

### 2.4. Ethical Consideration

First, the informed consent of hospital leaders was obtained before the questionnaire was distributed. The purpose and significance of the study were explained in detail to the participants, and informed consent was obtained. Second, we made sure that participants' responses were used only for the study and that they could opt out at any time. In addition, the study was conducted anonymously, did not include unethical practices or human clinical trials, and did not have any adverse effects on the physical or mental health of the participants. In the end, the Ethics Committee of Henan University approved the study (ID: HUSOM2022-238).

### 2.5. Statistical Analysis

Data were analyzed using SPSS 24.0 (Chicago, IL, USA) and AMOS 24.0 (IBM Corp.). The demographic characteristics of the participants were described using frequencies and percentages. The three main study variables (i.e., authentic leadership, conscientiousness, and voice behaviour) were described using means and standard deviations. An independent sample *t*-test or one-way analysis of variance (ANOVA) was used to identify differences in the study variable according to demographic characteristics. Pearson's correlation analysis was used to determine the correlation between the major study variables.

A two-step structural equation modeling (SEM) was used to test the hypotheses [27]. According to the recommendation of Hu and Bentler [28], the following goodness-of-fit statistics were used to evaluate the fit degree of model and data: the chi-square/degrees of freedom ratio (*χ*^2^/*df*), the root-mean-square error of approximation (RMSEA), goodness of fit (GFI), adjusted goodness of fit (AGFI), Tucker–Lewis index (TLI), comparative fit index (CFI), and root-mean- square residuals (RMR). A lower *χ*^2^/*df* value means better model fitting. Lower RMSEA also suggests better model fitting. GFI, TLI, CFI, AGFI, and NFI range from 0 to 1, with values closer to 1 indicating better fit. Finally, we used 5,000 samples for bootstrap resampling and a 95% confidence interval (CI) to test the direct and indirect effects between conscientiousness, authentic leadership, and voice behavior [29].

## 3. Results

### 3.1. The General Characteristics of the Subject

A total of 679 participants participated in the study. The participants were predominantly female (84.7%) and married (72.5%). Of the respondents, more than half had an age less than 40 years (74.3%) and had obtained a Bachelor's degree (69.7%). Regarding working experience, most study participants spent less than 10 years in the nursing profession. Moreover, Table 1 displays the differences in the basic characteristics of the participants in authentic leadership, Conscientiousness, and Voice behaviour. Nurse's perception of authentic leadership was significantly different in different gender, age, education, nursing tenure and monthly income. In terms of conscientiousness, there were significant differences in age, hiring method, and nursing tenure. Similarly, different education, hiring method, nursing tenure, and monthly income also significantly affected the score of voice behaviour (Table 1).

### 3.2. The Correlation of Variables

Pearson's correlation coefficient showed that there was a moderately positive correlation between authentic leadership and conscientiousness (*r* = 0.513, *P*  <  0.01) and voice behaviour (*r* = 0.632, *P*  <  0.01). Similarly, this relationship also existed between conscientiousness and voice behaviour (*r* = 0.630, *P*  <  0.01; Table 2).

### 3.3. Path Analysis of Each Variable

In order to explore the relationship between conscientiousness, authentic leadership, and voice behaviour of clinical nurses, voice behaviour was taken as the dependent variable, authentic leadership as the independent variable, and conscientiousness as the mediating variable. Based on the previous studies, a corresponding structural equation model was constructed, and the Bootstrap method was used for validation analysis. The model-fitting index was used to determine the fitness degree between the results and the data. The results showed that the model fitted well with the data (Table 3).

**Figure 1:**
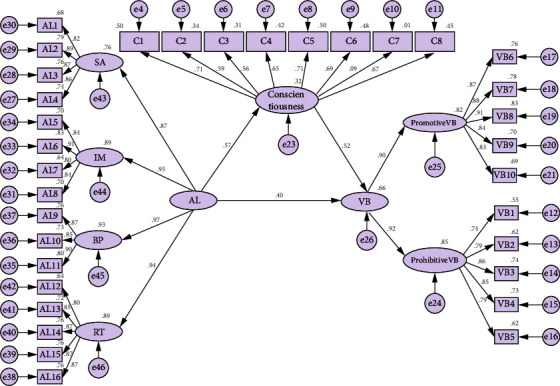
The structural model of this study. AL, authentic leadership; AL1-16, The 16 items of authentic leadership; SA, self-awareness; RT, relational transparency; IM, internalized moral; BP, balanced processing; C1-8, The 8 items of conscientiousness; VB, voice behaviour; Promotive VB, promotive voice behaviour; prohibitive VB, prohibitive voice behaviour; VB1-10, the 16 items of voice behaviour.

We conducted a path analysis to estimate the direct and indirect effects of both authentic leadership and conscientiousness on voice behaviour (Figure 1). The results revealed that authentic leadership has positive and significant direct effects on voice behaviour (*ß* = 0.396, *P* < 0.001) and conscientiousness (*ß* = 0.565, *P* < 0.001). Similarly, conscientiousness has a positive effect on voice behaviour (*ß* = 0.521, *P* < 0.001). When conscientiousness was added as the mediating variable, the effect size of authentic leadership on voice behaviour increased by 0.294, and the total effect size reached 0.691. Therefore, conscientiousness has a partial mediating effect between authentic leadership and voice behaviour (Table 4).

## 4. Discussion

This study was designed to determine the effect of authentic leadership on nurses' voice behaviour at work. And the structural equation model was used to confirm whether nurses' conscientiousness played a mediating role in the effect of authentic leadership on voice behaviour. The general characteristics show that 84.7% of the nurses that participated in this study were female, which is in line with previous studies performed in China [9].

In this study, a nurse' age, education, hiring method, length of clinical work experience, and monthly income are positively related to their voice behaviour. A previous study [30] found that the length of clinical working time and monthly income are important factors affecting nurses' voice behaviour, which was consistent with our research results. Our study found that clinical nurses who have worked for more than 15 years and earned more than 10,000 yuan per month scored higher in voice behaviour. This is consistent with the investigation of Zhao et al. [30]. Nurses who have worked for a short period of time are worried about their lack of professional knowledge and clinical experience, lack of mutual trust with leaders and colleagues, and fear of putting forward inappropriate opinions or suggestions, so nurses who have worked for a long time are more familiar with the work of the department and more willing to talk [31, 32]. In terms of education level, nurses with an associate's degree or below had more active voice behaviour. It may be because most of the highly educated nurses are new graduates. This was consistent with Wesche's research results which identified how newcomers will often choose silence to fit in (organizational socialization) rather than suggest changes which could risk relationships [33].

In addition, in this study, authentic leadership was positively correlated with nurses' voice behaviour, and authentic leadership had a positive and direct predictive effect on nurses' voice behaviour (*ß* = 0.396, *P* < 0.001). Voice aims at challenging the position of organizations, so it is risky [34]. Leaders usually regard the voices as threats, so they react negatively to outspoken employees [35]. Considering the risk of speaking, employees would carefully assess their social background before speaking [36]. If employees' voices are regarded as complaints or annoyances by leaders, they may face negative comments and be assigned to dissatisfying jobs [37]. At this time, employees would give up the voice to avoid adverse consequences and regard the voice as dangerous behaviour [38]. Authentic leaders know their own strengths and weaknesses and will be aware of their own limitations in making decisions and solving problems, so they are more likely to accept employees' ideas and opinions and even encourage employees to challenge the tradition. Furthermore, the authentic leader is committed to the establishment of a transparent relationship within the organization, which requires managers to balance the diversity and discrepancy of information. These acts will enhance subordinate trust in leadership, let them give up concerns, and allow them to express their true thoughts and feelings [39]. This shows that under the authentic leadership of the head nurse, the nurses and the head nurse trust each other and get closer to each other. When there is any problem in the work, the nurses will put the interests of the collective in the first place.

The results of this study showed that nurses' conscientiousness was a positive predictor of voice behaviour (*ß* = 0.521, *P* < 0.001), indicating that the higher the conscientiousness of nurses, the more positive their voice behaviour that is consistent with previous research [6]. Voice behaviour is the behaviour that employees take the initiative to make suggestions to leaders or point out the existing mistakes of the organization in order to improve the organizational effectiveness [40]. However, people with conscientiousness are generally more careful and prudent [41]. At the same time, people with high conscientiousness scores tend to be more goal-oriented [42]. In order to improve the organizational environment, they think they have a responsibility to contribute to the organization and are willing to have an active dialogue with colleagues and leaders [43]. They express their views patiently until they were understood [44].

The structural equation model analysis of this study showed that the direct predictive effect of head nurses' authentic leadership on nurses' voice behaviour was 0.396 (*P* < 0.001), the total effect of head nurses' authentic leadership on nurses' voice behaviour was 0.691 (*P* < 0.001) after adding conscientiousness personality, and conscientiousness had a partial mediating effect in the effect of head nurses' authentic leadership on nurses' voice behaviour. In other words, when nurses perceive the authentic leadership, their personality will affect their voice behaviour by their self-perception. This finding is in line with attribution theory, which holds that an individual's personality influences subsequent behaviour by judging and interpreting the behaviour of others or the external environment [15, 16]. Voice behaviour is aimed at promoting mutual cooperation, expressing employees' views and opinions on the organization. When the head nurse adopts an authentic leadership, nurses can feel the trust, care, and motivation from their superiors, so that they are more willing to integrate the development goals of the organization into their personal values and ideals [45]. Nurses with conscientious personality are cautious and responsible. Based on these characteristics, nurses with conscientiousness will not avoid talking about the problems found in their clinical work. Moreover, nurses with conscientiousness will be more likely to perceive and amplify the authenticity of the leaders [46], so as to promote the relationship between authentic leadership and voice behaviour.

## 5. Limitation

First of all, convenience sampling method was adopted in this study, and only a few hospitals in Henan Province were selected, with regional limitations, the generality and popularization of the conclusions may be limited. Second, the data obtained in this study were based on self-report. Although all participants had obtained anonymity and confidentiality, they still could not completely avoid reaction bias and social desirability bias could have affected the reporting of this behaviour. Finally, this was a cross-sectional survey, so the observed association should not be considered causal, and further research is required to explore the causal relationship.

## 6. Conclusion

This study explored the influence of authentic leadership and conscientiousness on the voice behaviour of nurses through the SEM. The results showed that authentic leadership and conscientiousness had a direct impact on voice behaviour. Conscientiousness partially mediated the relationship between authentic leadership and voice behaviour. Therefore, the nursing managers in the medical institutions, especially the head nurses, should actively develop an authentic leadership style. At the same time, training the conscientiousness of nurses can effectively promote voice behaviour. Since this study is a cross-sectional study, a longitudinal design is needed in the future to verify the validity of the findings.

## 7. Implication for Nurses' Management

Our findings provide many practical implications for hospital management. First of all, in view of the role of authentic leadership in promoting voice behaviour, the managers in medical institutions, especially the managers of nurses such as the head nurses, should actively change their leadership behaviour patterns and cultivate authentic leadership styles. Managers should share information, be open and honest in their dealings with employees, seek feedback from employees, involve them in decision-making, and demonstrate their ethical standards. Secondly, this study found that conscientiousness can play a partial mediating role between authentic leadership and voice behaviour, which suggests that managers should pay full attention to the fact that employees have individual differences. In their daily work, managers should identify employees' personality characteristics and formulate differentiated coping strategies in management. And managers also should pay attention to the selection and appointment of conscientious nurses. In order to arouse the enthusiasm of nurses, nurse managers can enhance their sense of responsibility by means of material, promotion, and modeling.

## Figures and Tables

**Table 1:** Participants' basic characteristics and differences in authentic leadership, conscientiousness, and voice behaviour (*n* = 679).

Variables	No. (%)	Authentic leadership		Conscientiousness		Voice behaviour	
		Mean (SD)	*t/F*	Mean (*SD*)	*t/F*	Mean (SD)	*t/F*
Gender^ǂ^			−2.015^*∗*^		−1.365		0.552
Male	111 (16.3)	3.87 ± 0.92		3.82 ± 0.57		3.89 ± 0.82	
Female	568 (84.7)	4.06 ± 0.74		3.89 ± 0.53		3.84 ± 0.76	
Age (years)^†^			3.245^*∗*^		5.426^*∗∗*^		4.164^*∗*^
<30	200 (29.5)	4.06 ± 0.76		3.78 ± 0.54		3.73 ± 0.72	
30–39	304 (44.8)	4.08 ± 0.79		3.94 ± 0.53		3.93 ± 0.79	
≥40	175 (25.7)	3.90 ± 0.74		3.90 ± 0.53		3.86 ± 0.76	
Marital status^†^			0.476		0.655		0.198
Single	174 (25.6)	4.06 ± 0.79		3.85 ± 0.52		3.82 ± 0.75	
Married	492 (72.5)	4.02 ± 0.77		3.89 ± 0.55		3.86 ± 0.76	
Others^a^	13 (1.9)	4.18 ± 0.81		3.98 ± 0.47		3.80 ± 1.03	
Education^†^			4.880^*∗∗*^		1.628		3.030^*∗*^
≤Associate's degree	165 (24.3)	4.13 ± 0.78		3.94 ± 0.55		3.95 ± 0.75	
Bachelor's degree	473 (69.7)	4.02 ± 0.76		3.86 ± 0.53		3.83 ± 0.77	
≥Master's degree	41 (6.0)	3.71 ± 0.82		3.84 ± 0.58		3.65 ± 0.74	
Hiring method^†^			1.866		4.559^*∗*^		5.481^*∗∗*^
Officially budgeted posts	216 (31.8)	3.96 ± 0.81		3.97 ± 0.56		3.98 ± 0.73	
Human resource agent	79 (11.6)	3.97 ± 0.78		3.88 ± 0.50		3.70 ± 0.77	
Independent contractor	384 (56.6)	4.08 ± 0.75		3.83 ± 0.53		3.81 ± 0.78	
Nursing tenure (years)^†^			2.895^*∗*^		3.348^*∗*^		5.019^*∗∗*^
≤5	214 (31.5)	4.11 ± 0.74		3.83 ± 0.54		3.83 ± 0.76	
6–10	171 (25.2)	4.00 ± 0.79		3.86 ± 0.54		3.87 ± 0.70	
11–15	160 (23.6)	3.91 ± 0.78		3.85 ± 0.52		3.72 ± 0.81	
≥15	134 (19.7)	4.12 ± 0.76		4.01 ± 0.53		4.05 ± 0.76	
Monthly income (¥)^†^			4.310^*∗∗*^		1.619		2.907^*∗*^
≤3000	38 (5.6)	3.85 ± 0.77		3.91 ± 0.53		3.65 ± 0.77	
3001–6000	270 (39.8)	3.96 ± 0.84		3.92 ± 0.53		3.82 ± 0.77	
6001–10000	304 (44.7)	4.13 ± 0.69		3.86 ± 0.55		3.92 ± 0.77	
≥10000	67 (9.9)	4.19 ± 0.71				3.98 ± 0.61	

^a^Others: divorced and widowed. ^ǂ^*t*-test for the independent group. ^†^One-way analysis of variance. ^*∗*^*P*  <  0.05; ^*∗∗*^*P*  <  0.01.

**Table 2:** Correlation between authentic leadership, conscientiousness and voice behaviour.

Variables	1	2	3
	*r* (*P*)		
(1) Authentic leadership	1		
(2) Conscientiousness	0.513^*∗∗*^	1	
(3) Voice behaviour	0.632^*∗∗*^	0.630^*∗∗*^	1

^*∗∗*^ *P*  <  0.01.

**Table 3:** Model-fitting standard and fitting index of the final model.

	*χ* ^2^/*df*	RMSEA	GFI	AGFI	TLI	CFI	RMR
Model-fitting standard	<3.0	<0.08	>0.9	>0.9	>0.9	>0.9	<0.08
Model-fitting index	2.994	0.054	0.887	0.859	0.941	0.945	0.035

*χ* ^2^/*df*, the chi-square/degrees of freedom ratio; RMSEA, root-mean- square error of approximation; GFI, goodness of fit; AGFI, adjusted goodness of fit; TLI, Tucker-Lewis; CFI, comparative fit index; RMR, root-mean-square residuals.

**Table 4:** Standardized direct, indirect and total effects of the hypothesized model.

	*ß*	SE	Percentile 95% CI		Bias-corrected percentile 95% CI		*P*
			Lower	Upper	Lower	Upper	
*Standardized direct effects*							
AL ⟶ Conscientiousness	0.565	0.036	0.490	0.631	0.489	0.630	<0.001
AL ⟶ VB	0.396	0.046	0.304	0.483	0.304	0.482	<0.001
Conscientiousness ⟶ VB	0.521	0.044	0.435	0.606	0.434	0.606	<0.001

*Standardized indirect effects*							
AL ⟶ VB	0.294	0.030	0.239	0.356	0.240	0.358	<0.001

*Standardized total effects*							
AL ⟶ Conscientiousness	0.565	0.036	0.490	0.631	0.489	0.630	<0.001
AL ⟶ VB	0.691	0.034	0.620	0.754	0.618	0.753	<0.001
Conscientiousness ⟶ VB	0.521	0.044	0.435	0.606	0.434	0.606	<0.001

*Note.* Standardized estimating of 5,000 bootstrap samples. AL, authentic leadership; VB, voice behavior.

## Data Availability

The [sav] data used to support the findings of this study are available from the corresponding author upon request.

## Abbreviations

ANOVA: Analysis of variance
SEM: Structural equation modeling
AL: Authentic leadership
AL1-16: The 16 items of authentic leadership
SA: Self-awareness
RT: Relational transparency
IM: Internalized moral
BP: Balanced processing
C1-8: The 8 items of conscientiousness
VB: Voice behaviour
Promotive VB: Promotive voice behaviour
Prohibitive VB: Prohibitive voice behaviour
VB1-10: The 16 items of voice behaviour.

## Additional Points

*Patient or Public Contribution*. Participants contributed to this study by completing a questionnaire that included sociodemographic data, authentic leadership, conscientiousness, and voice behaviour.
